# The child’s self-perception about dental decay in the change of deciduous teeth

**DOI:** 10.1080/07853890.2021.1897393

**Published:** 2021-09-28

**Authors:** Maria do Rosário Dias, Irene Ventura

**Affiliations:** ^a^Centro de Investigacão Multidisciplinar em Psicologia da Saúde, Centro de Investigação Interdisciplinar Egas Moniz (CiiEM), Egas Moniz Cooperativa de Ensino Superior, Caparica, Portugal

## Abstract

**Introduction:**

Currently, in Portugal, the percentage of children who have already been to an oral health appointment is 57.6% [1]. The prevalence of caries has declined over the years, with the expectation that by 2020, 59% of children will have a caries-free mouth. The present study aims to understand how the child experiences the Mental Representation of dental decay [2] and its implications on the self-perception inherent to the change of dentition (deciduous teeth).

**Materials and methods:**

In this exploratory study, the sample consists of 50 children of both genders, aged between 5 and 12 years, some have had prior contact with Dentist and others have not. A protocol was originally conceived and divided into five distinct sections: M1 and M2, where the child is asked to draw his self-portrait twice – before and after the loss of deciduous teeth; M3 where the child is asked to draw the mental representation of dental decay; M4, a set of six open-ended questions; M5, a sociodemographic questionnaire. The written answers were submitted to a content analysis grid that encompasses eight elementary analytical categories. The interpretation of the drawings [3] was carried out through a drawing content analysis grid, also designed for this purpose. Ethics authorisation was given by the institution (n°731/19.03.2019).

**Results:**

In the present poster only the preliminary results regarding the drawings collected in M3 will be presented crossed with the content analysis of the written narrative of question 5 – *What is dental decay for you?* (M4), as well as the data obtained in M5. The content analysis reveals remarkable differences according to the content analysis of the answers obtained in question 5. Out of the eight identified categories, the results obtained in the *bug* category (27.9%) followed by the *bacteria* category (18.9%) and *spot* (16.2%), seems to be in agreement with the fact that figures (M3) have been drawn which point to the symbolic representation of caries as a bug and/or *bacteria.*

**Discussion and conclusions:**

The results obtained seem to contribute to the (re) conceptualisation of the concept of caries in this age group when it is associated, also, the loss of deciduous teeth. The results also point to the need for developing educational tools for Oral Health Education.
